# Adult scoliosis and non-specific low back pain: analysis of trunk kinematics

**DOI:** 10.1186/1748-7161-8-S1-O32

**Published:** 2013-06-03

**Authors:** L Bissolotti, V Sani, M Gobbo, C Orizio

**Affiliations:** 1 Servizio di Recupero e Rieducazione Funzionale, Casa di Cura Domus Salutis, Brescia, Italy; 2 Sezione di Fisiologia Umana, Dipartimento di Scienze Biomediche e Biotecnologie, Università degli Studi di Brescia, Brescia, Italy; 3 LARIN: Laboratorio di Riabilitazione Neuromuscolare e Attività Fisica Adattata, Brescia, Italy

## Background

Adult scoliosis (AS) is an emerging issue in the field of spinal deformities management [1]. The increased prevalence results from the cumulative effect due to aging of patients affected by juvenile scoliosis (JS) plus the appearance of new cases in adult age.

## Aim

To provide data about trunk kinematics performance in patients with AS, and to compare it with non-specific low back pain (NL).

## Methods

Cotrel method was used to assess Cobb angle (CA) on plain x-ray. Bilateral trunk side bending (SB) and extension (TE) were evaluated with a two optoelectronic cameras (14markers, Gemini BTS spa, Milano, Italy)[2]. During active range of motion (aROM, °), speed of motion (SOM, °/sec) and error in trunk repositioning (ETR, °) were measured. Patients performed, as allowed by pain or discomfort, two movements for each direction.

## Results

AS-Group included 40 patients (10 men and 30 women, CA >15°, age 61.8±11.5 years, BMI 23.6±2.8kg/m2). A single curve was present in 32 patients (80%). CA of primary curve averaged 27.1±11.5° (range, 15–63°), thoracic CA averaged 25.5±22.3° (range, 8–58°). NL-Group included 40 patients, 9 men and 31 women (age was 58.2±10.9 years, BMI 23.9±3.2kg/m2). NL-Group averaged 35.7±12.3° in aROM on the right side, and 35.2±11.2° on the left (SOM 28.1±13.6°/sec) (p>0.05). AS-Group averaged 34.6±10.6° of aROM on the right side, and 35.5±12.5° on the left side (SOM 31.8±11.7°/sec) (p>0.05). Global trunk mobility during SB test averaged 71.0±21.2° in NL-group and 64.2±29.1° in AS-group (p>0.05), with no differences when considering the two different directions. During SB, 26% of the trunk aROM derived from the relative contribution of lumbar segment (L1-L5) (AS vs NL p>0.05). TE averaged 23.7±8.1° in NL-Group, (L1-L5: 54.5±26.3%) and 22.6±8.1° in AS-Group (L1-L5: 60.8±30.6%) (p>0.05). NL group ETR was 3.4±2.7° during SB and 3.6±2.0° during TE (p>0.05). In AS group, ETR was 3.4±1.5° during SB and 2.9±2.0° during TE (p>0.05).

## Conclusions

In an AS-Group of patients, the kinematic performance, and the ability to control spinal motion (SOM and ETR), was similar to a NL-Group. Mild to moderate scoliosis is not influencing the motor control of the spine. As previously shown in NL[3], physiotherapy programs for AS do not require more attention in trunk proprioception.

## References

[B1] Aebi M The adult scoliosis Eur Spine J 2005 14 10 925 948 10.1007/s00586-005-1053-9 16328223

[B2] Gombatto SP Collins DR Sahrmann SA Engsberg JR Van Dillen LR Patterns of lumbar region movement during trunk lateral bending in 2 subgroups of people with low back pain Phys Ther 2007 87 4 441 454 10.2522/ptj.20050370 17374634

[B3] Lee AS Cholewicki J Reeves NP Zazulak BT Mysliwiec LW Comparison of trunk proprioception between patients with low back pain and healthy controls Arch Phys Med Rehabil 91 9 1327 1331 20801248 10.1016/j.apmr.2010.06.004 PMC4896302

